# Outcomes and Follow-Up Trends in Adrenal Leiomyosarcoma: A Comprehensive Literature Review and Case Report

**DOI:** 10.3390/jcm13123499

**Published:** 2024-06-14

**Authors:** Federico Maria Mongardini, Maddalena Paolicelli, Antonio Catauro, Alessandra Conzo, Luigi Flagiello, Giusiana Nesta, Rosetta Esposito, Andrea Ronchi, Alessandro Romano, Renato Patrone, Ludovico Docimo, Giovanni Conzo

**Affiliations:** 1 Division of General, Oncological, Mini-Invasive and Obesity Surgery, University of Study of Campania “Luigi Vanvitelli”, 80138 Naples, Italy; madd.paolicelli@gmail.com (M.P.); antonio.catauro@unicampania.it (A.C.); aleconzo@hotmail.it (A.C.); luigi.flagiello1@studenti.unicampania.it (L.F.); giusiana.nesta@gmail.com (G.N.); rosetta.esposito@unicampania.it (R.E.); alessandroromano1995@gmail.com (A.R.); ludovico.docimo@unicampania.it (L.D.); giovanni.conzo@unicampania.it (G.C.); 2 Division of Pathology, Department of Mental Health and Preventive Medicine, Luigi Vanvitelli University of Campania, 80138 Naples, Italy; andrea.ronchi@unicampania.it; 3 Division of Epatobiliary Surgical Oncology, Istituto Nazionale Tumori IRCCS Fondazione Pascale-IRCCS di Napoli, 80131 Naples, Italy; renato.patrone@istitutotumori.na.it

**Keywords:** adrenal tumor, leiomyosarcomas, mesenchymal tumor

## Abstract

**Background**: Leiomyosarcoma (LMS) originating from the adrenal gland is exceedingly rare, constituting a minute fraction of soft tissue sarcomas. Due to its rarity, with less than 50 documented cases in English medical literature, the diagnosis and management of adrenal LMS remain challenging. The aim of this study was to perform a review of the literature, in order to evaluate the prognosis of these rare cancers and report our specific case. **Methods**: A systematic review of the literature was conducted using PubMed, Web of Science, Google Scholar, and Scopus databases, up to December 2020. The search utilized MeSH terms such as “Adrenal Gland Neoplasms,” “Leiomyosarcoma,” “Adrenalectomy,” and “Smooth Muscle Tumor.” The inclusion criteria focused on studies reporting patients with a histopathological diagnosis of adrenal leiomyosarcoma. The PRISMA guidelines were followed to ensure a comprehensive analysis. **Results**: Out of 63 identified studies, 43 met the inclusion criteria and were reviewed. These studies highlighted the rarity and aggressive behavior of adrenal leiomyosarcoma. Surgical excision remains the cornerstone of treatment, often complemented by adjuvant therapies. The reviewed case involved a 52-year-old woman who underwent a right laparoscopic adrenalectomy for a 9 × 7 × 6 cm grade 3 leiomyosarcoma. Despite subsequent adjuvant chemotherapy, hepatic metastases were detected, illustrating the aggressive nature of the disease. The literature underscores the importance of histopathological analysis and long-term surveillance for managing disease progression. **Conclusions**: Optimal management of adrenal leiomyosarcoma requires a multidisciplinary approach and meticulous follow-up. The rarity of the disease poses challenges for standardizing treatment, but surgical excision and tailored adjuvant therapies show promise. Further research is essential to refine treatment strategies and improve prognosis for this rare malignancy.

## 1. Introduction

Leiomyosarcoma (LMS) is a mesenchymal tumor that originates from smooth muscle cells [1], representing approximately 25% of all soft tissue sarcomas (STS), a heterogeneous group of neoplasms of mesenchymal origin. Among these, adrenal leiomyosarcoma is exceedingly rare, posing significant challenges in diagnosis and management due to its infrequent occurrence and aggressive nature.

Tumors are located more frequently in the retroperitoneum or intra-abdominal region (35% of cases), while those originating in the uterus account for 30% of leiomyosarcomas [2]. A recent review of case reports in the English medical literature revealed fewer than 50 instances documented [3]. Here, we present a case report focusing on a rare instance of adrenal leiomyosarcoma, emphasizing surgical management with laparoscopic adrenalectomy (LA). In fact, this study aims to bridge this knowledge gap by reviewing the existing literature and integrating findings. The case is noteworthy not only for its rarity but also for the diagnostic complexity and the therapeutic approaches it necessitated, including the onset of hepatic metastases post-surgery.

In addition to the primary adrenal lesion, our case report highlights the occurrence of hepatic metastases, indicating the aggressive nature of adrenal leiomyosarcoma and its propensity for systemic spread. This dissemination underscores the necessity for comprehensive treatment approaches, including surgical intervention and adjuvant chemotherapy, to address both the primary tumor and its metastatic deposits. Furthermore, the presence of hepatic metastases further emphasizes the importance of vigilant surveillance and multidisciplinary management to optimize patient outcomes in the face of this challenging disease. Through this comprehensive examination, the manuscript endeavors to enhance the oncological community’s understanding of adrenal LMS, promote early diagnosis, and refine therapeutic strategies to improve prognosis for this rare disease.

## 2. Case Report

A 52-year-old patient was referred to the General Surgery Division of the University of Campania Luigi Vanvitelli (Naples, Italy) in September 2022 due to the presence of a solid neoformation in the right adrenal gland. The patient presented, in July of the same year, with symptoms including tachycardia, hypertensive crisis, dyspnea, oppressive chest pain, general discomfort, and cramping pain in the abdomen or right flank region. Initial investigations did not reveal significant pathologies. Preoperative laboratory tests assessing adrenal function revealed no significant abnormalities. Plasma and urinary levels of catecholamines (adrenaline, noradrenaline, dopamine) and metanephrines were within normal ranges. Both plasma and urinary cortisol levels were normal, as were aldosterone and renin levels. Adrenal steroids, including dehydroepiandrosterone sulfate (DHEAS), were also likely within normal limits. Abdominal ultrasound showed a solid formation measuring 70 mm adjacent to the inferior vena cava in the right adrenal lodge and retroperitoneal site, with normal kidneys bilaterally.

**Figure 1:**
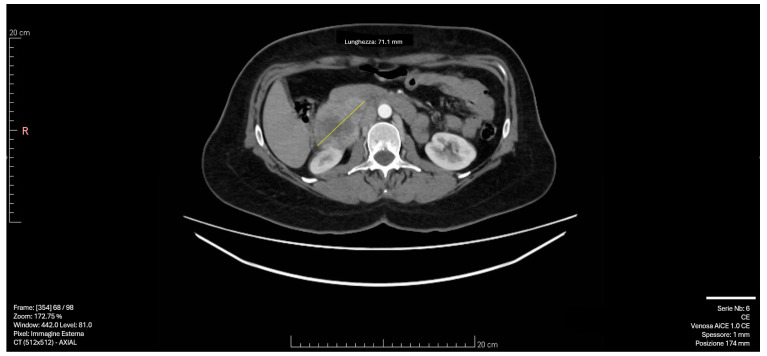
CT scan image: The right adrenal mass demonstrated adjacency to but no infiltration of the inferior vena cava, with venous drainage observed in the right ovarian vein. The yellow arrow indicates the 55 × 50 mm solid formation with a necrotic core and irregular peripheral enhancement in the right adrenal lodge.

Subsequent CT imaging with intravenous contrast revealed a 55 × 50 mm solid formation with a necrotic core and irregular peripheral enhancement in the right adrenal lodge. The mass demonstrated adjacency to but no infiltration of the inferior vena cava, with venous drainage observed in the right ovarian vein. The left adrenal gland appeared normal, with no significant lymphadenopathy [Figure 1].

**Figure 2:**
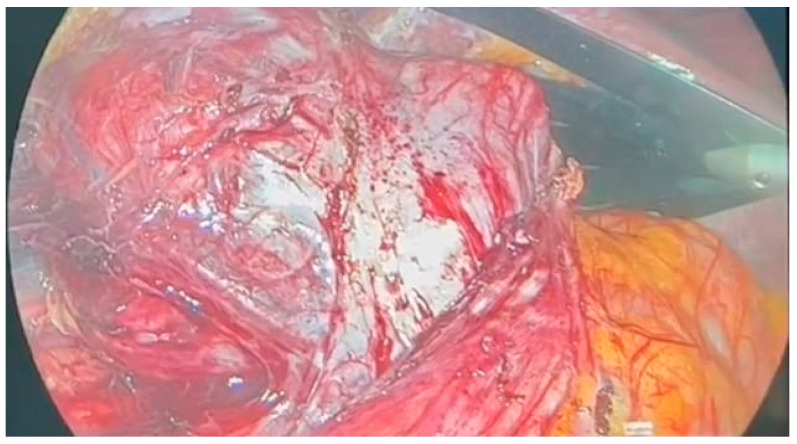
Intraoperative adrenal mass.

Following multidisciplinary evaluation, a right adrenalectomy was recommended. The laparoscopic procedure was performed under general anesthesia, with pneumoperitoneum initiated using Hasson’s trocar in the paraumbilical area. The highly vascular adrenal mass was densely adhered to the inferior vena cava, exerting external compression on the ipsilateral ureter and kidney, resulting in kidney hypotrophy and complete dislocation [Figure 2].

**Figure 3:**
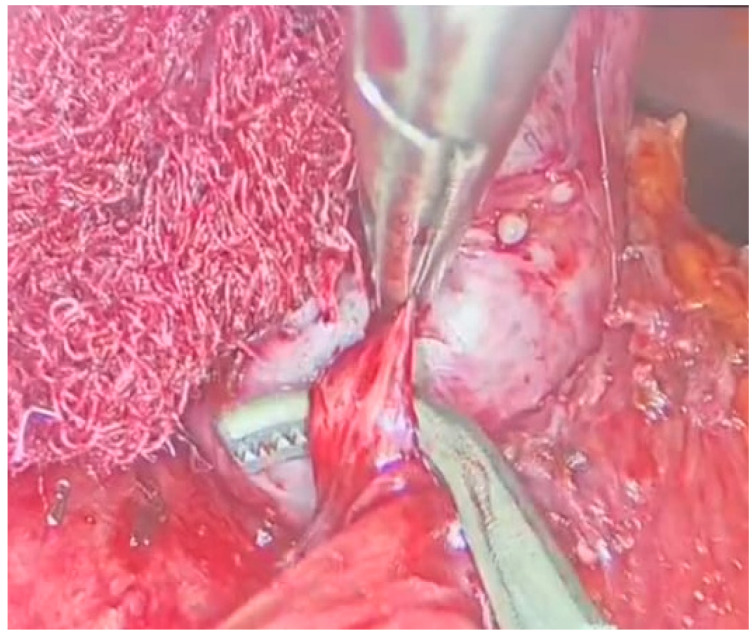
Laparoscopic adrenalectomy, dissection from the residual adhesions.

With meticulous dissection and the use of a radiofrequency instrument, the mass was successfully freed from adhesions [Figure 3]. Afferent vessels were ligated and sectioned, and the mass was completely excised using an endobag.

Careful hemostasis was achieved, and Hemopatch^®^ and Floseal^®^ were placed. Grossly, the specimen was constituted by a solid nodular mass of 9 × 7 × 6 cm and weighing 148 g, with a grayish cut surface.

**Figure 4:**
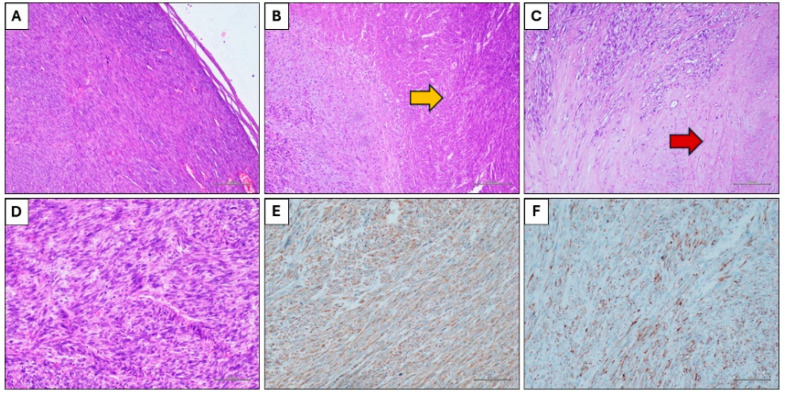
Histological findings. (**A**) Spindle cells neoplasms with expansive growth (Hematoxylin and eosin stain, original magnification 100×). (**B**) Coagulative necrosis (yellow arrow) (Hematoxylin and eosin stain, original magnification 100×). (**C**) Hyalinization of the stroma (red arrow) (Hematoxylin and eosin stain, original magnification 100×). (**D**) Neoplastic cells with nuclear atypia and mitotic figures (Hematoxylin and eosin stain, original magnification 200×). (**E**) Immunohistochemical positivity for smooth muscle actin (Immunohistochemical stain, original magnification 100×). (**F**) Immunohistochemical positivity for desmin (Immunohistochemical stain, original magnification 100×).

Histological examination showed a solid neoplastic proliferation characterized by an expansive growth and constituted by spindle cells arranged in a fascicular pattern (Figure 4A). Some extensive areas of coagulative necrosis (Figure 4B) were present, as well as areas of hyalinization of the stroma (Figure 4C). The neoplastic population was constituted by spindle cells with hyperchromic and irregular nuclei, and frequent mitotic figures (Figure 4D). Immunohistochemistry showed positivity for smooth muscle actin (Figure 4E), desmin (Figure 4F) and calponin, and negativity for cytokeratin, S100, EMA.

A final diagnosis of grade 3 leiomyosarcoma was rendered.

The patient resumed oral feeding the day after surgery and was discharged after 4 days with a complete resolution of symptoms. After careful multidisciplinary evaluation with various dedicated specialists, a decision was made for close and careful follow-up, pending further imaging tests such as CT or PET-CT at 4 months to continue the therapeutic process.

**Figure 5:**
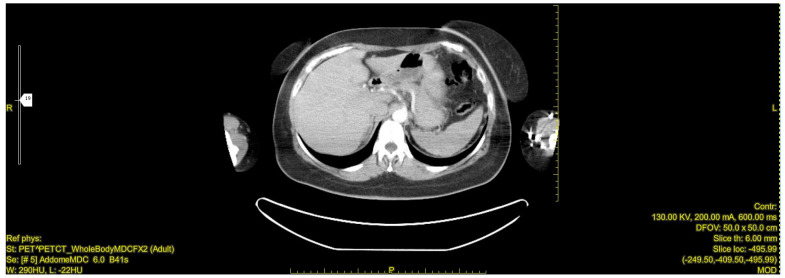
Postoperative Abdominal CT: Non postoperative complications at the surgical site.

**Figure 6:**
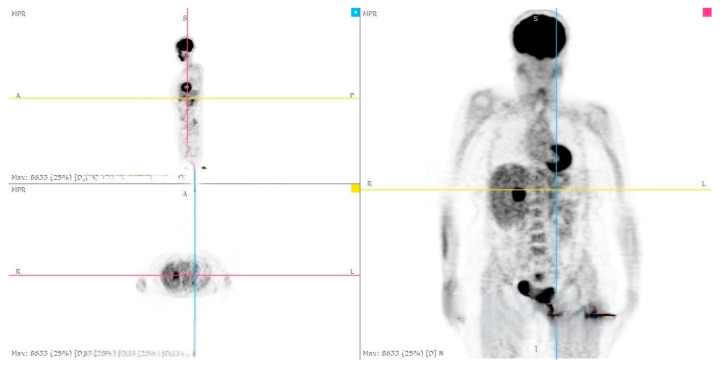
PET-CT whole body: hepatic metastasis with a SUV of 14.3.

**Figure 7:**
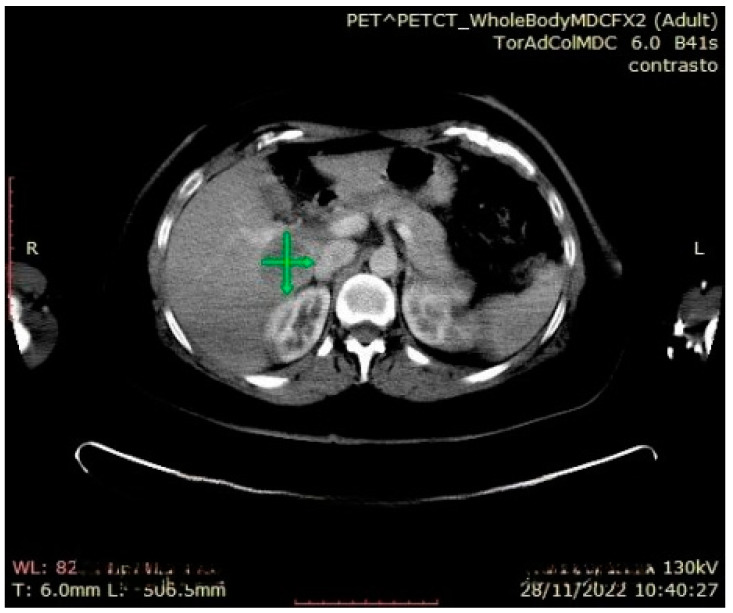
Abdominal CT: hepatic metastasis identified with green arrows.

During the follow-up examination at 4 months post-surgery, a PET-CT scan without contrast conducted at another facility revealed a prominent focal hypermetabolic lesion in the hepatic segment V measuring approximately 51 × 44 mm with a SUV of 14.3, indicative of a secondary lesion. Additionally, osteolytic lesions were observed along the lateral aspect of the third rib on the right hemithorax (SUV max 3.5) and the inferior angle of the left scapula (SUV max 3.2), along with multiple bilateral pulmonary nodules (SUV max 1.2) [Figure 5, Figure 6 and Figure 7]. Hence, the aggressive metastatic dissemination of leiomyosarcoma underscores the imperative for a prompt reassessment of the treatment approach and the contemplation of intensified therapeutic interventions.

In accordance with the described hepatic metastasis presentation, the patient underwent chemotherapy treatment consisting of 9 cycles of Epirubicin + Dacarbazine followed by an additional 3 cycles with Dacarbazine alone.

The follow-up proceeded with the monitoring of symptoms and objective findings for 12 months post-surgical treatment, actively monitoring the secondary manifestations identified in the PET-CT with biochemical and instrumental evaluation, and in constant and diligent multidisciplinary collaboration.

## 3. Methods

Using the PubMed, Web of Science, Google Scholar and Scopus databases, a systematic review of the current literature was carried out, up to January 2000. The MeSH (Medical Subject Headings) search terms used were: “Adrenal Gland Neoplasms”, “Leyomiosarcoma”, “Adrenalectomy”, “Smooth Muscle Tumor”.

**Figure 8:**
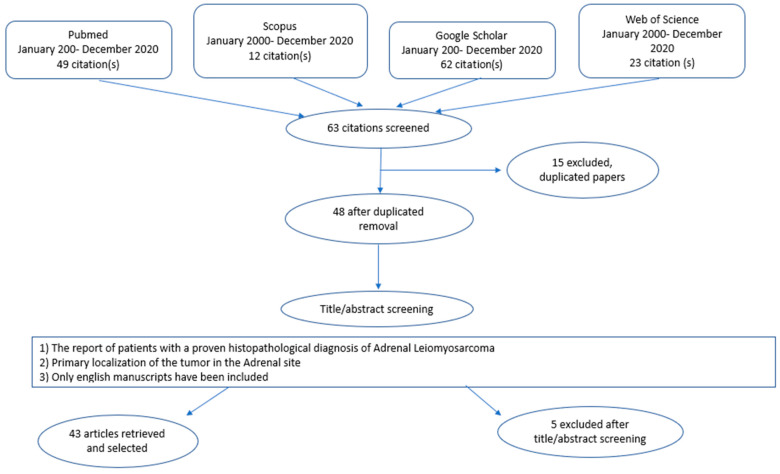
PRISMA Statement: Flow-Chart. Diagram of the systematic review of the iterate performed in 4 databases from January 2000 up to December 2020. Search terms included: adrenal tumor, Leiomyosarcomas, mesenchymal tumor”. Inclusion criteria are shown in the central box. Major reasons for exclusion were duplicated papers from the different databases (n = 15), the language of the manuscripts included (n = 4). Further reasons for exclusion were primary location of the tumor in the Adrenal site (n = 1). This led to the final selection of 43 studies which fulfilled the inclusion criteria.

The authors observed that adrenal LMS was an extremely rare neoplasm. The keywords “Adrenal Gland”, “Leiomyosarcomas”, “Mesenchymal Tumors” were used for the research. Several combinations of the keywords and MeSH terms were utilized as showed: “Adrenal Leiomyosarcomas”, “Mesenchymal tumors”. The various terms were substituted during the search. References of the more relevant articles were manually searched. The last research was concluded in December 2020. The search was carried out by two authors, FMM and MP, and the obtained results were discussed with the senior author GC. The final article was realized in accordance with the Preferred Reporting Items for Systematic Reviews and Meta-Analyses (PRISMA Statement) guidelines, and was not registered in any systematic review registry [Figure 8] [4]. The following data were extracted from the included studies: first author, year of publication, publishing journal, characteristics of study population, potential combination of surgical and chemoradiotherapeutic treatment, and follow-up (in months).

The inclusion criteria of the study comprised the reports of patients with a proven histopathological diagnosis of adrenal leiomyosarcoma, with primary localization of the tumor in the adrenal site. All studies that failed to fulfil the established inclusion criteria, and the non English language studies, were excluded. In all the studies, adrenal LMS diagnosis was based on the definitive pathology.

The clinical characteristics included age, sex, localization of the neoplasm, size (in centimeters).

## 4. Results

Sixty-three suitable studies were identified after the literature review. After the removal of a duplicate study, forty-eight articles were selected for the full-text review. Four studies were excluded because there were in Spanish [5,6,7], and in Korean [8]. Another one was ruled out because the primary tumor was not primary localized in the adrenal gland, but as a secondary metastasis, and also because it was in Japanese [9]. Therefore, forty-three met our inclusion criteria and were enrolled in the current review. The features of the forty-three selected studies were summarized in Table 1.

### 4.1. Demographic and Clinicopathological Features

The data revealed that adrenal neoplasms showed no strong preference for any specific age group or sex, with cases reported in individuals ranging from young adolescents to the elderly, and an equal distribution between males and females. This diversity emphasized the importance of including adrenal gland tumors in differential diagnoses for all age groups and both genders.

From the selected studies, forty-five patients with a histological diagnosis of adrenal leiomyosarcoma were identified (24 females and 21 males). The mean age was 51.18 ± 15.26 years, with a median age of 60 ± 15.23 years, ranging from 14 to 78 years. The mean age in female cases was approximately 56.88 with a standard deviation of 14.60, and the median was 60.5 with a standard deviation of 14.60, ranging from 14 to 78 years. In male cases, the mean age was approximately 51.62 with a standard deviation of 14.76, while the median was 50 with a standard deviation of 14.76, ranging from 29 to 75 years. In 21 cases, the tumor was located on the left with one case of multiple metastases, two cases of adjacent organ invasion, one case of renal vein extension, one of inferior vena cava extension, and another with extension of the inferior vena cava and both iliac veins. For three left adrenal leiomyosarcomas, the extent was not specified, and in 12 cases, there was no extension to other organs. Twenty-two adrenal leiomyosarcomas were located on the right, with four cases of inferior vena cava invasion, two cases of inferior vena cava and right atrium invasion, three cases of adjacent organ invasion, one case of pulmonary metastasis and aorta invasion, one case of multiple metastases, and in 3 studies, extension was not specified. In seven cases, there was no extension to other organs. In two cases, the tumor was bilateral without extension to other organs. The mean size of the specimen was 9.35 ± 5.45 cm, but 4 studies did not report the size.

Regarding treatment options, one case underwent adrenalectomy associated with partial nephrectomy without adjuvant chemo-radiotherapy and survived 12 months without recurrence or metastasis [10]. In six cases, adrenalectomy associated with nephrectomy was performed [10,11,17,23,25,26]. Specifically, three cases had no adjuvant therapy [17,25,26] and a mean survival of 10.33 ± 1.79 months without recurrence or metastasis; one case received adjuvant chemoradiotherapy [11], and another one received only adjuvant radiotherapy [23], both alive with metastasis at 9 months follow-up. Also in our study, laparoscopic adrenalectomy and nephrectomy were performed, followed by adjuvant chemotherapy, and the patient was alive with liver metastasis at 12 months follow-up.

In 12 cases, adrenalectomy was performed with thrombectomy [15,19,20,24,48], cavotomy IVC [34,42,48], radiofrequency ablation [28], partial diaphragmatic resection [31], liver partial resection and lymphadenectomy [42,43], distal pancreatectomy with splenectomy [51], hepatic lobectomy and cholecystectomy [16] due to extension to other organs. In 9 of these cases, there was no adjuvant therapy, with 6 patients dying from metastasis at 1 and 12 months follow-up. Two patients were alive without recurrence at 10 months, one patient had a recurrence at 3 months, and in one study, there was no information about follow-up. In 4 cases, extended surgery was followed by adjuvant therapy, with one patient dying shortly after, one patient dying with metastasis at 16 months, one patient alive with metastasis at 6 months, and one patient without follow-up data.

In 20 cases, only adrenalectomy was performed, with one case being bilateral [18]. Our case report and the other four from the literature were laparoscopic [1,40,41,44], within one case converted to open surgery [44]. In 15 cases, adrenalectomy was not followed by adjuvant therapy; furthermore, in three of these cases follow-up data were not available, while the other twelve patients were alive without recurrence or metastasis at a mean follow-up time of 16.91 ± 7.75 months. In one study, exploratory laparotomy was performed without adjuvant therapy, resulting in death after 3 weeks [14]. In one case, palliative chemotherapy and radiotherapy were administered without follow-up information [47]. In one cases, patients underwent chemotherapy, resulting in death at 3 months and survival with metastasis at 9 months, respectively [29,36]. In one case, the patient was treated with radiotherapy and died 11 days later with metastasis [27].

### 4.2. Definitive Pathology Examination and Immunohistochemistry

The pathology examination and immunohistochemistry findings of various studies on spindle cell tumors exhibited several commonalities and differences [Table 2].

Macroscopically, many of these tumors demonstrated central areas of cystic degeneration and necrosis. This central necrosis was a common feature observed in some studies [10,11,15,24], among others. Hemorrhage was also frequently associated with these necrotic areas, as seen in the studies by Lack et al. and Lujan et al. [11,16], indicating vascular involvement and rapid growth of the tumors. However, there were differences in the descriptions of the masses, with some studies reporting multinodular appearances, fibrous substrates, and varying weights, such as the 2400 g mass reported by Candanedo-Gonzalez et al. [21] and the 180 g mass described by Mohanty et al. [23]. In 14 studies, macroscopic characteristics were not described [5,12,13,17,20,22,27,28,29,30,41,43,45,47].

Morphologically, the most consistent feature across these studies was the presence of spindle-shaped cells arranged in fascicles, as consistently reported by Choi et al., Lack et al., Matsui et al., Wang et al., Aoki et al. and Lee et al. [10,11,15,22,24,45], suggesting a mesenchymal origin or differentiation of these tumors. These cells arranged in interlacing fascicles, a pattern observed in studies by Matsui et al., Shao et al., and Zhou et al. [15,30,40]. Atypia and pleomorphism were also common features, indicating the malignant potential and variability in tumor cell morphology. For instance, Lack et al. and Mohanty et al. [11,23] described spindle cells with blunt-ended nuclei and eosinophilic cytoplasm; otherwise, Kato K et al. [19] reported pleomorphic neoplastic cells.

Furthermore, eight reports, including those by Candanedo-Gonzalez et al., Mohanty et al., and Deshmukh et al., described pleomorphic neoplastic cells [16,21,23,32,34,42,46]. High cellularity and nuclear atypia were notable features in studies by Lee et al., Manzano et al., and Mulani et al. [19,47,52]. Candanedo-Gonzalez et al. and Wang et al. described osteoclast-like giant cells [21,24]. Eight studies did not mention the microscopic cellular characteristics [1,12,13,17,20,27,43]. Eight studies did not complete the microscopic cellular information at all, not mentioning mitotic activity either [18,22,28,29,30,39,45,48].

Mitotic activity was another area with significant findings. High mitotic rates were regularly reported, reflecting the aggressive nature of these tumors. Specific figures vary, with Lack et al. reporting 15 mitoses per 10 high power fields (HPFs) and Mohanty et al. noting 12–14 mitoses per HPF, while Lee et al. observed up to 25 mitotic figures per 10 HPFs [11,22,23]. Other studies, such as Deshmukh et al. and Li et al., noted mitotic rates ranging from 10–12/10 HPFs to 18/10 HPFs [32,43]. The Ki-67 proliferation index, which indicates the percentage of tumor cells undergoing mitosis, also varies widely. Lujan et al. reported a Ki-67 index of about 80%, while Gulpinar et al. noted a lower index of 4%, indicating the variability in the proliferative capacities of these tumors [16,33].

Immunohistochemical analysis revealed a consistent profile for adrenal spindle cell neoplasms, which allows for the molecular diagnosis of adrenal leiomyosarcoma. Smooth muscle actin (SMA) was universally positive across studies, including those by Lack et al., Goto et al., and Bhalla et al. [11,25,36]. This positivity was evident in studies by Lack et al., Etten et al., Linos et al., and Mohanty et al., indicating the smooth muscle differentiation of the spindle cell tumors [11,14,16,18]. Desmin positivity was observed in our case and also in 24 reports [1,19,21,22,23,24,26,29,30,32,34,35,36,37,38,39,40,41,43,46,49,50,51,53], while it was negative in three cases [18,31,44]. Vimentin, a marker for mesenchymal origin, was frequently positive, as seen in the studies by Lack et al., Kato et al., Sakellariou et al., Tzaida et al. and Deshmukh et al. [11,19,32,46,50]. On the other hand, markers such as S-100, CD34, and cytokeratins were often negative, helping to exclude other tumor types like neural, endothelial, and epithelial neoplasms. This negativity was consistently reported in studies by Kato et al., Mohanty et al., and Wei et al. [19,23,37]. Seven studies did not explicitly mention immunohistochemistry results [5,10,13,16,17,20,27].

## 5. Discussion

Adrenal leiomyosarcoma is a rare malignant tumor originating from smooth muscle cells within the adrenal gland [54]. This malignancy is characterized by its complex molecular underpinnings and the signaling pathways implicated in its development. Despite its rarity, the diagnosis and management of this aggressive tumor present a quite challenging [17,20]. The comprehensive analysis of leiomyosarcoma incidence and treatment in adrenal glands necessitate a meticulous examination of individual data points in relation to the aggregate findings, ensuring robust conclusions. Our study’s bibliographic data, meticulously compiled from various sources, serves as a crucial foundation for understanding the landscape of this rare malignancy. The literature on adrenal gland neoplasms from the analyzed cases presents a complex picture of the challenges involved in treating this rare but significant cancer type [1,5,10,11,12,13,14,15,16,18,19,20,21,22,23,24,25,26,27,28,29,31,32,33,34,35,36,37,38,39,40,41,42,43,44,45,46,47,48,49,50,51,55]. The variability in tumor characteristics, the need for sometimes extensive surgical intervention, and the potential for adjunct treatments such as chemotherapy and radiation therapy all contribute to the diverse outcomes observed in these patients. This review highlights the heterogeneity in the presentation and management of adrenal gland neoplasms. The variability in tumor characteristics and the consequent choice of treatment significantly impact patient outcomes.

### 5.1. Demographic Features

Analyzing the intersection of variables such as age, sex, tumor size, side, and extension, it is clear that each factor plays a crucial role in determining the approach to surgical and adjuvant chemoradiation therapies, as well as influencing follow-up outcomes in patients treated for adrenal-related conditions.

Younger patients might be candidates for more aggressive treatments, including extensive surgeries and adjuvant therapies, given their longer expected lifespan and potentially better ability to recover from intense treatments. Conversely, in older patients, clinicians might opt for more conservative treatments, balancing the benefits of aggressive therapy against potential risks and the overall health status of the patient. The age of the patient also impacts follow-up strategies, with more frequent monitoring possibly required for those who undergo aggressive treatments at a younger age to detect recurrences early. Sex may not directly influence the choice of treatment modalities but plays an indirect role through physiological and metabolic differences between males and females that can affect how treatments are tolerated and their effectiveness. These differences might also necessitate tailored follow-up regimens to address sex-specific risks or complications post-treatment. Larger tumors often require more radical surgical approaches, possibly combined with chemotherapy or radiotherapy, to manage the greater risk of local spread or metastasis. In contrast, smaller tumors might be managed with less invasive procedures, relying on the slow-growing nature of some adrenal tumors. The initial size of the tumor heavily influences follow-up outcomes; larger tumors may have a higher risk of recurrence, requiring longer and more intensive monitoring to manage potential complications effectively.

Tumor side (left, right, or bilateral) can affect surgical planning, especially considering the anatomical challenges and potential complications associated with operating on either or both sides of the body. Bilateral tumors may necessitate a more complex and cautious approach, impacting both the immediate and long-term management plans.

The extension of the tumor beyond the adrenal gland into surrounding structures or distant sites significantly impacts the choice of treatment. Tumors with extensive spread are likely treated with combination therapies—surgery to remove as much of the tumor mass as possible, followed by radiation or chemotherapy to address residual disease. The extent of disease at diagnosis is a strong predictor of follow-up needs, as patients with a more extensive disease at the outset may require more rigorous and prolonged surveillance to detect recurrence or manage ongoing issues.

In summary, the interaction of these factors—age, sex, tumor size, side, and extension—shapes the therapeutic approach and follow-up care in managing adrenal-related conditions. Understanding these relationships helps clinicians tailor treatments to individual patient profiles, aiming for the best possible outcomes while minimizing risks and side effects. Aggressive management, including extensive surgery and adjunctive therapies, appears crucial for managing advanced cases.

Case reports provide valuable insights into its clinical presentation, diagnostic challenges, and therapeutic outcomes. Zhou et al. and Wei et al. contributed to the literature with a comprehensive literature review, emphasizing the importance of integrating clinical, radiological, and histopathological findings for accurate diagnosis and optimal treatment planning [5,25,29,31,37,40,43,46,47,56]. Zetler et al. reported a case in an AIDS patient, suggesting a potential link between immunocompromised states and leiomyosarcoma [12]. The rarity of adrenal leiomyosarcoma, particularly in pediatric populations, is exemplified by cases such as the laparoscopic excision of a bilateral primary adrenal leiomyosarcoma in a 14-year-old girl with AIDS, reported by Linos et al. [18]. In fact, ALMS cases have been reported in association with immunocompromised states such as AIDS, suggesting a possible link to Epstein–Barr virus infection, as indicated by Zetler and Boman et al. [12,13]. This underscores the importance of considering the underlying conditions in disease management and follow-up planning. Furthermore, Nagaraj et al. expanded demographic understanding, with a rare case in an Arab male, while Nerli et al. highlighted challenges in young adults [39,49].

However, the optimal treatment strategy should be tailored to individual patient characteristics, considering the invasiveness of the tumor and the patient’s overall health condition.

### 5.2. Histological Findings

Regarding the histological findings and immunohistochemistry, spindle cell tumors commonly show a spindle-shaped cell morphology, high mitotic activity, and positive immunoreactivity for smooth muscle markers. There are variations in the specific details of cell morphology, mitotic rates, and the expression of additional immunohistochemical markers, reflecting the heterogeneity within this group of tumors. These findings highlight both the shared characteristics and the unique aspects of each case, providing a comprehensive understanding of spindle cell tumors’ pathological and immunohistochemical profiles.

### 5.3. Molecular Bases and Target Therapy

Its molecular basis involves intricate genetic and epigenetic changes, frequently including alterations in the RB1, TP53, and PTEN genes, which are fundamental to the regulation of the cell cycle, apoptosis, and cellular proliferation [57]. Epigenetic modifications also play a central role in PAL’s pathogenesis, which can lead to the activation of oncogenes and the suppression of tumor suppressor genes, thereby promoting tumor growth and progression [58]. The involvement of microRNAs (miRNAs) in PAL is another significant aspect. The dysregulation of specific miRNAs has been linked to various malignancies, including leiomyosarcomas: In PAL, particular miRNAs may act either as oncogenes or tumor suppressors, though the exact miRNAs involved are still under investigation [59].

Additionally, the tumor microenvironment, which includes interactions with surrounding stromal cells and the extracellular matrix, plays a critical role in influencing the behavior of PAL. These interactions can facilitate tumor growth, angiogenesis, and metastasis through various signaling pathways, such as those mediated by TGF-beta, VEGF, and PDGF [60].

To develop targeted therapies effectively, a deep understanding of these molecular bases is essential. Although challenging, advancements in molecular biology and genetics offer hope for more effective treatment approaches. Ongoing research into the molecular mechanisms governing PAL is crucial for the development of personalized medical strategies and improving patient outcomes with this rare malignancy [61]. Targeted therapies are designed to exploit specific genetic and molecular characteristics of the tumor cells, offering a more precise approach than conventional treatments.

Tyrosine kinase inhibitors (TKIs) have shown promise in treating soft tissue sarcomas due to their ability to interfere with key signaling pathways that promote tumor growth and survival. Drugs such as pazopanib, a multi-targeted TKI, have been approved for advanced soft tissue sarcoma after positive outcomes in clinical trials [62]. These inhibitors target vascular endothelial growth factor receptors (VEGFRs), which are often implicated in the angiogenesis associated with tumor growth.

The mammalian target of the rapamycin (mTOR) pathway is another critical pathway in the progression of various cancers, including LMS. mTOR inhibitors, such as sirolimus and everolimus, have been studied for their effectiveness in slowing down tumor growth by inhibiting cell proliferation and inducing apoptosis [63]. Although results have been mixed, these agents offer a potential treatment avenue, particularly in tumors resistant to conventional chemotherapy.

Monoclonal antibodies targeting growth factor receptors and other tumor-associated antigens are under investigation for LMS. Trabectedin, a drug initially derived from a marine organism, has shown efficacy in LMS by binding to the minor groove of DNA and disrupting the transcription of oncogenes [64].

Recent advances have also focused on targeting the specific genetic mutations and epigenetic alterations found in LMS cells. Agents that modify epigenetic marks, such as HDAC inhibitors, are being explored to re-activate tumor suppressor genes and inhibit oncogenes. These therapies are still largely in the experimental stages, but represent a significant step toward personalized medicine [65,66].

Although in its infancy for LMS, immunotherapy approaches, including checkpoint inhibitors, are being evaluated in clinical trials. These treatments aim to boost the body’s immune response against tumor cells. Early studies suggest a variable response, likely dependent on the immunogenicity of the tumor [67,68].

While the development of targeted therapies for leiomyosarcoma, including PAL, is challenging due to the tumor’s rarity and molecular complexity, ongoing research into these therapies holds significant promise. These targeted treatments aim to improve survival rates and quality of life by tailoring interventions to the specific molecular profiles of leiomyosarcoma tumors.

### 5.4. Surgical Management

The surgical management of primary adrenal leiomyosarcoma through adrenalectomy is a cornerstone in the treatment of this rare malignancy. It involves various approaches, including laparoscopic adrenalectomy, which has been successfully employed in selected cases. Quildrian et al. reported a case of primary adrenal leiomyosarcoma treated with laparoscopic adrenalectomy, highlighting the feasibility and efficacy of minimally invasive techniques in managing this rare malignancy [41]. Various surgical approaches have been described in the literature, including radical excision, adrenalectomy, and even vena cava resection in cases of tumor invasion, as illustrated by Ozturk et al. [34]. Wang et al. proposed a novel approach to the surgical resection of leiomyosarcoma involving the adrenal vein, emphasizing the importance of meticulous surgical planning and technique [24]. The surgical management of adrenal leiomyosarcoma poses significant challenges due to the tumor’s rare occurrence and aggressive behavior. Among the pivotal decisions in surgical oncology is the choice between open and laparoscopic adrenalectomy; each approach has distinct advantages and considerations that influence treatment outcomes, especially in complex cases like adrenal leiomyosarcoma. The open approach is particularly advantageous in cases where tumors are suspected to invade adjacent organs or the inferior vena cava, as it allows for a more radical resection and the ability to manage intraoperative complications directly [53,69]. Conversely, laparoscopic techniques also provide an enhanced visualization of the surgical field, which can be beneficial in the meticulous dissection required to preserve adrenal function in non-invasive tumors [52,70]. In the last few decades, minimally invasive transperitoneal laparoscopic adrenalectomy has become the standard for the surgical resection of adrenal gland tumors [71].

However, minimally invasive retroperitoneal adrenalectomy has gained popularity as an alternative technique, offering shorter hospital stays, reduced postoperative pain, fewer complications, and improved cosmetic outcomes [22,72,73]. However, the laparoscopic approach requires significant expertise and is limited by the size and invasiveness of the tumor. It is generally preferred for tumors smaller than 6 cm that do not show signs of local invasion. The precision of laparoscopic instruments facilitates careful manipulation and dissection around the adrenal glands, reducing the risk of damaging major vessels [55].

An accurate preoperative examination is essential for selecting eligible patients for laparoscopic adrenalectomy, but also an operative team composed of experienced and skilled surgeons is necessary [30,72,74]. Choi and Liu described the angiographic features of adrenal leiomyosarcoma, providing valuable insights into the vascular characteristics of these tumors that can aid in preoperative planning and intraoperative decision-making [10]. The low risk associated with adrenalectomy, particularly via a laparoscopic approach, provides a definitive diagnosis and treatment without significant risk or cost [75,76].

Studies comparing these approaches show varied results; however, the trend suggests that laparoscopic adrenalectomy can be safely performed with comparable outcomes to open surgery in terms of oncological efficacy when conducted by experienced surgeons. A systematic review highlights that laparoscopic resection for small, localized adrenal leiomyosarcomas achieves outcomes similar to the open approach, with the added benefits of a minimally invasive procedure [77]. Ongoing advances in surgical technology, such as the integration of robotic systems, may further refine the laparoscopic approach, expanding its applicability to more complex adrenal surgeries. Further research and accumulation of case-specific outcomes are necessary to develop clearer guidelines that assist in choosing the most appropriate surgical approach for adrenal leiomyosarcoma [78]. Advanced energy devices, when used responsibly, can enhance surgical outcomes, ensuring cost savings and patient satisfaction [79,80]. The choice of hemostatic device is based on surgeon preference; in our experience, we used a hemostatic flap [74,81]. In conclusion, while laparoscopic adrenalectomy offers significant advantages in selected cases, the choice of surgical approach must be individualized based on a thorough assessment of the tumor’s characteristics and the patient’s overall condition. This tailored approach ensures optimal surgical outcomes and adherence to oncological principles.

### 5.5. Adjuvant Therapy and Follow-Up

Furthermore, the role of adjuvant therapies, such as chemotherapy and radiotherapy, remain controversial due to the lack of standardized treatment protocols and limited evidence from clinical trials [26,36]. However, in the cases of unresectable or metastatic diseases, systemic chemotherapy may be considered to palliate symptoms and prolong survival, as suggested by Bhalla et al. [36]. Aoki et al. reported a case of primary adrenal leiomyosarcoma in an elderly woman, where the tumor was managed surgically without adjuvant therapy; this was similarly described by Lokanatha et al., where surgery alone was employed for a primary adrenal leiomyosarcoma [45,51].

Lack et al. conducted an immunohistochemical and ultrastructural study of a primary leiomyosarcoma of the adrenal gland, elucidating the histopathological features and immunophenotypic profile of these tumors, which are crucial for definitive diagnosis and prognostication [11,16]. Various histological subtypes of adrenal leiomyosarcoma have been described, including pleomorphic leiomyosarcoma and leiomyosarcoma with osteoclast-like giant cells, underscoring the histopathological heterogeneity of this malignancy [21,23,44]. Histopathological insights from Deshmukh and Gulpinar et al. aid accurate diagnosis, guiding treatment decisions [32,33]. Lee et al. showcased histological diversity, underlining the need for a nuanced pathological evaluation [35].

Regular surveillance is imperative for monitoring disease recurrence, metastasis, and treatment response following surgical resection [28]. Imaging modalities such as 18F-FDG-PET, as highlighted by Van Laarhoven et al., play a crucial role in detecting the early signs of disease progression and guiding subsequent management decisions [27].

A long-term follow-up is essential in the management of adrenal leiomyosarcoma to monitor for recurrence and metastasis [38]. In cases of adrenal leiomyosarcoma with extension into adjacent structures, such as the right atrium and inferior vena cava, highlighting the importance of vigilant surveillance and timely intervention in cases of advanced disease [14,15,19,48]. Such instances highlight the need for vigilance in surveillance imaging and clinical assessment to detect metastatic spread promptly. Moreover, the heterogeneity of ALMS behavior is exemplified by Nerli et al., where despite aggressive treatment, metastasis and adverse outcomes can occur [49]. Conversely, other cases, such as those reported by Nagaraj et al., demonstrate favorable long-term outcomes with appropriate management [39].

In cases of metastatic disease or lymph node involvement, the management becomes even more complex. Onishi et al. reported a case of primary adrenal leiomyosarcoma with lymph node metastasis, highlighting the importance of thorough staging and consideration of systemic therapies [42,82].

The overmentioned chemotherapeutic regimen with Epirubicin + Dacarbazine was selected to address the aggressive metastatic behavior of the leiomyosarcoma, targeting both the hepatic and extrahepatic sites of the involvement identified by imaging. The decision to administer sequential chemotherapy cycles reflects the ongoing effort to optimize disease control and improve the patient’s overall prognosis in the face of advanced disease progression [83].

As evidenced by the case reports gleaned from the scientific literature regarding adrenal leiomyosarcoma, there is an overall average of 10 months of follow-up among all cases reported in Table 1. Specifically, out of 44 cases, survival without metastasis was observed in 17 cases [5,10,12,15,17,24,25,26,30,32,33,35,37,40,41,44,46], survival with metastasis or recurrence in 10 cases [11,21,22,23,34,36,38,43,50] (including our case report), and death occurred in 10 cases, with one case in the perioperative period [16] and the remaining cases during postoperative follow-up [14,15,19,20,27,28,29,45,48] (ranging from 3 weeks to 16 months). Nine patients were lost to follow-up [1,13,18,31,39,47,49,51].

Adrenal leiomyosarcoma exhibits considerable mortality and invasiveness, posing significant challenges in clinical management and prognosis. Many studies have highlighted its aggressive nature and poor outcomes despite therapeutic interventions [84].

### 5.6. Strengths and Limits

This study utilizes a review of existing literature combined with a detailed case report, offering a unique dual perspective on the rare condition of adrenal leiomyosarcoma. The methodology employed rigorous data collection from established databases, ensuring a robust analysis of available data. We employed multidisciplinary evaluations in our case report, illustrating the complex nature of diagnosis and management in adrenal leiomyosarcoma.

However, the rarity of the disease limits the ability to perform a large-scale, randomized controlled trial and therefore may affect the generalizability of the results. The retrospective nature of the literature review may introduce selection bias, as cases documented in the literature might not be representative of all real-world scenarios.

## 6. Conclusions

These cases underscore the importance of multidisciplinary collaboration, meticulous surgical technique, and accurate diagnosis in optimizing outcomes for patients with primary adrenal leiomyosarcoma. A correct and precise preoperative diagnosis is very difficult and challenging in most cases. The extensive variability in tumor characteristics and patient outcomes revealed through our review indicates that the successful management of ALMS requires a nuanced approach that integrates advanced surgical techniques and, where appropriate, targeted adjuvant therapies. Despite the challenges posed by its rarity and aggressive nature, ongoing advancements in molecular understanding and surgical methodologies provide a beacon of hope for improving treatment strategies and patient survival rates.

Surgical resection remains the cornerstone of treatment, with the choice between laparoscopic and open adrenalectomy being tailored to the tumor’s specifics and patient condition. The potential of targeted therapies, such as tyrosine kinase inhibitors and mTOR pathway inhibitors, is promising, reflecting a shift towards more personalized treatment plans based on the genetic and molecular landscape of the tumor. Furthermore, regular and vigilant follow-up is crucial for monitoring disease recurrence and progression, which is pivotal in adjusting management strategies promptly.

In conclusion, while the challenges in managing adrenal leiomyosarcoma are formidable, a multidisciplinary approach that leverages the latest research and clinical innovations holds the key to optimizing outcomes. By continuing to refine surgical techniques and explore new therapeutic avenues, there is potential to significantly enhance both the quality of life and the survival rates for patients afflicted with this formidable cancer.

## Figures and Tables

**Table 1:** Literature review of the 44 existing cases (and our case report) of adrenal leiomyosarcoma, including subsequent surgical treatment and follow-up strategies.

References	Age/y	Sex	Side	Size (cm)	Extension	Surgery	CHT/RT	Follow-Up/Months
CHOI SH, J Surg Oncol 1981 [10]	50	F	L	16	None	Adrenalectomy + Partial Nephrectomy	None	12 (alive without recurrence or metastasis)
LACK EE, Am J Surg Pathol 1991 [11]	49	M	R	11	None	Adrenalectomy + Nephrectomy	RT + CHT	9 (alive with metastasis)
ZETLER PJ, Arch Pathol Lab Med 1995 [12]	30	M	L	11	ND	Adrenalectomy	None	20 (alive without recurrence or metastasis)
BOMAN F, Arch Pathol Lab Med 1997 [13]	48	M	R	2	ND	None	None	ND
	29	M	L	0.8	ND	None	None	ND
ETTEN B, Sarcoma 2001 [14]	73	F	R	27	IVC	Exploratory Laparotomy	None	3 weeks (dead)
MATSUI Y, Int J Urol 2002 [15]	61	F	R	ND	IVC + right atrium	Adrenalectomy + Nephrectomy + Thrombectomy	None	1 (dead with metastasis)
LUJAN MG, Arch Pathol Lab Med 2003 [16]	63	M	R	25	Pulmonary metastasis and Invasion to AO	Adrenalectomy + Nephrectomy + hepatic lobectomy + cholecystectomy	CT	died shortly after surgery
THAMBOO TP, Pathology 2003 [17]	68	F	R	13	None	Adrenalectomy + Nephrectomy	None	12 (alive without recurrence or metastasis)
LINOS D, Surgery 2004 [18]	14	F	Bil	3.5 (R) 4 (L)	None	Bilateral Adrenalectomy	None	ND
KATO K, Int J Clin Oncol 2004 [19]	59	M	L	10	IVC	Adrenalectomy + Nephrectomy + Thrombectomy	None	6 (dead with metastasis)
WONG, J R Soc Med 2005 [20]	57	M	L	ND	IVC and both iliac veins	Adrenalectomy + Nephrectomy + Thrombectomy	None	more than 6 months (dead with recurrence)
CANDANEDO-GONZALEZ FA, Endocr Pathol 2005 [21]	59	F	L	16	Invasion to Adjacent Organs	Adrenalectomy	RT + CHT	36 (alive with metastasis)
LEE CU, Abdom Imaging 2006 [22]	49	M	L	3	None	Adrenalectomy	None	10 (alive without recurrence or metastasis)
MOHANTY SK, Urology 2007 [23]	47	F	L	10	None	Adrenalectomy + Nephrectomy	RT	9 (alive with metastasis)
WANG TS, World J Surg Oncol 2007 [24]	64	F	R	14	IVC + right atrium	Adrenalectomy + Thrombectomy	None	10 (alive without recurrence or metastasis)
GOTO J, Endocr J 2008 [25]	73	F	R	8	Invasion to Adjacent Organs	Adrenalectomy + Nephrectomy	None	10 (alive without recurrence or metastasis)
MENCOBONI M, Clin Med Oncol 2008 [26]	75	F	R	8	None	Adrenalectomy + Nephrectomy	None	12 (alive without recurrence or metastasis)
VAN LAARHOVEN HV, Anticancer Res 2009 [27]	78	M	L	ND	multiple metastasis	None	RT	11 days (dead with metastasis)
HAMADA S, Int J Clin Oncol 2009 [28]	62	F	Bil	8 (R) 4 (L)	None	Bilateral Adrenalectomy, Radiofrequency Ablation	RT + CHT	16 (dead with metastasis)
KARAOSMANOGLU A, J Ultrasound Med 2010 [29]	63	M	R	ND	IVC	None	CT	3 (dead)
SHAO IH, Chang Gung Med J 2012 [30]	66	M	L	10	Renal vein	Adrenalectomy	None	18 (alive without recurrence or metastasis)
KANTHAN R, World J Surg Oncol 2012 [31]	28	F	L	16	None	Adrenalectomy + Nephrectomy + Partial Diaphragmatic	None	ND
DESHMUKH SD, J Cancer Res Ther 2013 [32]	60	F	L	5	None	Adrenalectomy	None	21 (alive without recurrence or metastasis)
GULPINAR MT, Case Rep Urol 2014 [33]	48	M	r	11	None	Adrenalectomy	None	8 (alive without recurrence or metastasis)
OZTURC H, Rare Tumors 2014 [34]	70	F	R	8	IVC	Adrenalectomy + Cavotomy	CT	6 (alive with metastasis)
LEE S, Korean J Pathol 2014 [35]	28	M	R	15	None	Adrenalectomy	None	18 (alive without recurrence or metastasis)
BHALLA A, Conn Med 2014 [36]	45	M	R	11	multiple metastasis	None	CT	9 (alive with metastasis)
WEI J, Int J Surg Pathol 2014 [37]	57	F	L	8	None	Adrenalectomy	None	29 (alive without recurrence or metastasis)
MANZANO AJ, AACE Clin Case ReP 2015 [38]	63	F	R	6.8 × 4.4	ND	Adrenalectomy	CT	2 (local recurrence, treated with CT)
NAGARAJ V, Case Rep Surg 2015 [39]	61	M	L	16 × 10.7 × 11.7	None	Adrenalectomy	None	NA—lost contact with the patient
ZHOU Y, Int J Clin Exp Pathol 2015 [40]	49	F	L	6 × 5 × 5	None	Laparoscopic Adrenalectomy	None	6 (alive without recurrence or metastasis)
QUILDRIAN S, Endocrinol Nutr. 2015 [41]	44	F	R	7.4 × 5.2	None	Laparoscopic Adrenalectomy	None	3 years (alive without recurrence or metastasis)
ONISHI T, World J Surg Oncol. 2016 [42]	34	M	R	5.2 × 3.2	IVC	Adrenalectomy en bloc with VIIs of liver, lymphadenectomy, resection of invaded IVC wall	None	10 (alive without recurrence or metastasis)
LI C-C, Urol Sci. 2016 [43]	61	F	R	4 × 5	Invasion to Adjacent Organs	Adrenalectomy with resection of invaded hepatic tissue	None	3 (tumor recurrences)
RUDIN A, Clin Surg. 2016 [44]	72	M	L	5.1	None	Laparoscopic converted to open Adrenalectomy	None	3 (alive without recurrence or metastasis)
AOKI C, J Clin Case Rep 2017 [45]	81	F	R	7	None	Adrenalectomy	CT	2–4 local recurrences, 8–10 further growth of tumor; 13 died.
TZAIDA O, Ann Clin Exp Metabol 2017 [46]	69	F	R	5.4 × 4.3	ND	Adrenalectomy	RT	12 (alive without recurrence or metastasis)
MULANI SR, Case Rep Gastrointest Med 2018 [47]	50	M	L	8.1	ND	NONE	PALLIATION CT-RT	NA
DOPPALAPUDI SK, BMJ Case Rep CP. 2019 [48]	70	M	R	12.2	IVC, Invasion to Adjacent Organs	Adrenalectomy + Nephrectomy + IVC Cavotomy + Thrombectomy	None	12 (pulmonary metastasis, died)
NERLI R, Int Cancer Confer J. 2020 [49]	27	M	L	9 × 6.5 × 7	None	Adrenalectomy	None	NA
SAKELLARIOU M, Molecular and Clinical Oncology [50]	62	M	L	10.3 × 8.5 × 8.4	Invasion to Adjacent Organs	Adrenalectomy	CT-RT	3 (bone metastasis), 7 (liver metastasis), 11 (pulmonary metastasis), 31 (alive with metastasis)
LOKANATHA Indian J Med Paediatr Oncol. 2020 [51]	60	F	L	7.6 × 7.7 × 6.8	None	Adrenalectomy + distal pancreatectomy, splenectomy	CT	NA
Waack A, Radiol Case Rep. 2022 [1]	58	F	L	5.5 × 4.4	None	Laparoscopic Adrenalectomy	None	NA
Our Case Rep	52	F	R	9 × 7 × 6	None	Laparoscopic Adrenalectomy + Nephrectomy	CT	12 (alive with liver metastasis)

IVC: Inferior Vena Cava.

**Table 2:** Definitive pathology examination and immunohistochemistry.

References	Macroscopic	Cell Morphological Characteristics	Mitotic Activity	Immunohistochemistry
CHOI SH, J Surg Oncol 1981 [10]	Central area of cystic degeneration and necrosis	spindle-shaped cells arranged in bundles atypia with nuclear hyperchromatism.	evident	-
LACK EE, Am J Surg Pathol 1991 [11]	Necrotic and hemorrhagic area of neoplasm	spindle cells having elongated, blunt-ended nuclei and tapering, eosinophilic cytoplasmic processes	15 per 10 high power fields of mitotic figures	Vimentin ab +; S-100 protein −/+; HHF-35 +; α-SMA +.
ZETLER PJ, Arch Pathol Lab Med 1995 [12]	-	-	-	SMA +
BOMAN F, Arch Pathol Lab Med 1997 [13]	-	-	-	-
	-	-	-	-
ETTEN B, Sarcoma 2001 [14]	areas of coagulation necrosis	spindle cell tumor with a moderate degree of atypia	up to 10 mitoses per 2 mm^2^	strong immunoreactivity for α-SMA
MATSUI Y, Int J Urol 2002 [15]	central area of cystic degeneration	interlacing fascicles of spindle cells	many mitoses	cytoplasmic (SMA) immunoreactivity
LUJAN MG, Arch Pathol Lab Med 2003 [16]	hemorrhagic, multinodular mass with irregular zones of necrosis	pleomorphic neoplastic cells	Ki-67 about 80%	
THAMBOO TP, Pathology 2003 [17]				
LINOS D, Surgery 2004 [18]	fibrous substrate, with inflammatory areas	characteristic spindle-shaped neoplastic cells	-	Positive for smooth muscle actin, vimentin, and actin HHF Negative for S100, CD 34, desmin, and ALK.
KATO K, Int J Clin Oncol 2004 [19]	areas of hemorrhage and necrosis	spindle-shaped cells with a fibrillary appearance	several mitotic figures	Positive for SMA, desmin and vimentin. Negative for neuron-specific enolase (NSE), S-100, chromogranin, synaptophysin, and CD34
WONG, J R Soc Med 2005 [20]	-	-	-	-
CANDANEDO-GONZALEZ FA, Endocr Pathol 2005 [21]	a circumscribed, solid, multinodular, green-red mass, with markedly necrotic and hemorrhagic areas, and extra adrenal extension 2400 g	hypercellular areas, with a diffuse proliferation of pleomorphic neoplastic cells and osteoclast-like giant cells	15 mitotic figures per 10 high power fields, Ki-67 was 7.6%	Positive for SMA, desmin vimentin and p53, Tumor cells were negative for CD34, HMB45, estrogen receptors, and S-100 protein.
LEE CU, Abdom Imaging 2006 [22]	-	hypercellular tumor with a haphazard arrangement of spindled, oval, or rounded cells intermingled with bizarre tumor cells	-	positive for desmin and negative for creatine kinase, myoglobin, S-100, CD34, CD117 (Ckit), and HMB-45
MOHANTY SK, Urology 2007 [23]	It was almost completely replaced by a centrally hemorrhagic and partially necrotic rubbery to firm mass 180 g	neoplastic cells were spindled to plump, with cigar-shaped blunt-ended nuclei. In areas, the tumor showed moderate-to-marked nuclear pleomorphism and conspicuous nucleoli.	brisk mitotic activity (12–14/HPFs) ki67 was > 75%	strong immunoreactivity for muscle markers (desmin, calponin, and actin), whereas those for cytokeratins (pankeratin, AE1/AE2), renal cell markers (RCC, CD10), melanoma markers (S100, melan A), and adrenal markers (Inhibin, melan A) were negative
WANG TS, World J Surg Oncol 2007 [24]	a firm, nodular and trabeculated tan-white mass with focal areas of cystic degeneration and hemorrhage Areas of coagulative necrosis were present	composed of spindle cells arranged in intersecting fascicles. Tumor cells showed moderate atypia with hyperchromasia, nuclear enlargement and occasional giant cells.	three to five mitoses per 10 high power fields were identified, with occasional atypical mitotic figures	strong reactivity for desmin and smooth muscle actin; negative for c-kit, S100 and HMB45
GOTO J, Endocr J 2008 [25]	White rubbery tissue with central degeneration due to necrosis and hemorrhage	Fascicles of spindle cells	Mitotic rate as high as 2–5 cells/high power field (HPF)	Positive for SMA; CD57, S-100 and CD117 were negative, the composed cells were massively positive with NSE protein
MENCOBONI M, Clin Med Oncol 2008 [26]	A roundish, fibrous neoformation with a thrombus inside the adrenal vein	Elongated cells with eosinophilic cytoplasm and elongated nuclei with rounded and small nucleolus with moderate polymorphism, arranged in fascicles, no necrotic areas	16/50 HPF mitotes Ki67 20%	Positive for desmin, SMA(1A4), Actin (HHF3, HHF35, Primary Antibody 5); CD34, CD117 were negative.
VAN LAARHOVEN HV, Anticancer Res 2009 [27]	-	-	-	-
HAMADA S, Int J Clin Oncol 2009 [28]	-	Spindle- shaped neoplastic cells		Strong staining of SMA, but negative staining for keratin, CD34 e c-kit
KARAOSMANOGLU A, J Ultrasound Med 2010 [29]		Spindle cells sarcoma with smooth muscle differentiation consistent with high grade leiomyosarcoma		Diffuse staining for actin and vimentin and patchy staining for desmin and keratin
SHAO IH, Chang Gung Med J 2012 [30]		Spindle cells tumor, composed of interlacing fascicles of neoplastic smooth muscle cells		Positive for desmin and SMA, negative for CD117, HMB45, CD34.
KANTHAN R, World J Surg Oncol 2012 [31]	Ragged, irregular, pale rubbery multinodular mass with hemorrhage and necrosis 1492 g	Tumor cells arranged in sheets and storiform patterns with extensive fibrous spindled stroma in some areas and others with a myxoid background	Ki67 and p53 moderately expressed	Strongly positive with SMA and vimentin, negative staining to pan-keratin, CK7, CK20 and HMWK. Negative staining with myogenin and desmin, S-100 was negative too.
DESHMUKH SD, J Cancer Res Ther 2013 [32]	Uniform, grey-white color with streaks and strands and a whorled appearance in places; no areas of hemorrhage and necrosis	Spindled neoplastic cells, arranging in interlacing bundles and fascicles of varying sizes.	10 to 12 abnormal mitotic figures per 10 HPFs	Strong reactivity for SMA, desmin and vimentin; negative for CK, S-100 protein, CD117, HMB45 and CD34
GULPINAR MT, Case Rep Urol 2014 [33]	Roundish fibrose neoplasia, yellow-creamy-white solid mass with a central necrotic area. 370 g	Spindle cells associated with predominantly diffuse plasma cell infiltration prevalent in the necrotic areas	Ki67 was 4%	Strongly diffuses vimentin, moderately diffuses SMA; negative for pan-cytokeratin, inhibin, synaptofisin, chromogranin, CD30-31-34-117, S-100, ALK, myogenin, lambda an k light chain proteins immunonegative
OZTURC H, Rare Tumors 2014 [34]	Cystic features and bleeding at focal point, no necrosis	Cells with spindle-shaped nuclei and pink cytoplasm that crossed each other at varying angles and pleomorphism at certain foci	8–10/10 HPFs, ki67 was 70%	SMA, desmin positive; CK and bcl2 negative
LEE S, Korean J Pathol 2014 [35]	Enlarged lobulate mass of 792 g, well sub-circumscribed and partially encapsulated tumors	Spindle cells tumor with a rim of fibrous tissue with entrapped atrophic adrenal cortical cells, geographic coagulation necrosis with surrounding fibrosis	25/10 HPFs	Strongly positive for SMA and desmin; pan cytokeratin, CD117, S-100 protein and HMB45 were all negative
BHALLA A, Conn Med 2014 [36]		Atypical spindle-shaped cells arranged in intersecting fascicles. Focal tumor necrosis	High mitotic activity	SMA and desmin were positive, S-100 and c-kit were negative
WEI J, Int J Surg Pathol 2014 [37]	Firm, grayish white mass surrounded by a fibrous pseudo capsule, with focal areas of hemorrhage	Large area of necrosis, neoplastic cells arranged in interlacing fascicles; in some areas it ranged from spindled to plump with cigar-shaped, blunt-ended nuclei	More than 10 abnormal mitotic figures per 50 HPFs	Strongly positive for SMA, desmin and vimentin; negative for cytokeratin, CD117, CD34, CD68, S-100, HMB45, myoglobin
MANZANO AJ, AACE Clin Case ReP 2015 [38]	A reconstructed mass with heterogeneous borders and areas of internal necrosis	Spindle cells neoplasia, atypical mitosis and necrosis	Ki67 focally greater than 50%, present in 40% of proliferating cells	Positive for SMA, desmin and focal myogenin (MYF4); S-100 and inhibin were negative
NAGARAJ V, Case Rep Surg 2015 [39]	Grossly, multiple pieces of grayish white friable tissue with attached small amount of fatty tissue, with mucoidal areas and tiny hemorrhagic areas. 1040 g	High-grade spindle cells with eosinophilic cytoplasm and spindly, blunt ended vesicular nuclei	-	Strongly positive for desmin and vimentin; negative for calretinin, inhibin, chromogranin, synaptophysin, S-100 pankeratin and CD68.
ZHOU Y, Int J Clin Exp Pathol 2015 [40]	Roundish, grayish white mass, with areas of hemorrhage and necrosis	Hypercellular tumor, intersecting fascicles of spindle cells	Ki67 > 60%	Positive for desmin, SMA, vimentin and negative for CD34, S100, CD117, bcl2 and Dog1.
PEREIRA-BECEIRO J, Cir Esp 2015 [5]				
QUILDRIAN S, Endocrinol Nutr. 2015 [41]	-	Interlacing fascicles of spindle cells with elongated hyperchromatic nuclei and marked pleomorphism	Ki67 was 20%, with up to 12 mitoses/10 HPFs	Strongly positive for vimentin (80%), H caldhemon (90%), SMA (70%), desmin (10%), actin HHF-35 (80%), CD34 (60%); CD117-68, S100, cytokeratin AE1 AE3 were negative.
ONISHI T, World J Surg Oncol. 2016 [42]	Solid and grayish white mass with some mucoid and small necrotic areas	Spindle-shaped cells with eosinophilic cytoplasm and pleomorphic nuclei	Ki67 approximately 50% in the hot spot	Diffusely positive for SMA and negative for S100 and CD34
LI C-C, Urol Sci. 2016 [43]	-	-	18/10 HPFs	Positive for SMA, desmin, H caldesmon; negative for S100, CD34
RUDIN A, Clin Surg. 2016 [44]	White lobulated fleshy area that focally grew into the lumen of the adrenal vein, red and hemorrhagic soft area	Intersecting fascicles of spindle cells with atypical cigar-shaped nuclei and abundant eosinophilic cytoplasm, not necrosis area	Scattered mitotic figures were present	Strongly positive for SMA; negative for desmin, HMB45, MITF, inhibin
AOKI C, J Clin Case Rep 2017 [45]	-	Atypical spindle cells with oval-shaped nuclei growing in a flowing sequence	-	Positive SMA and vimentin;
TZAIDA O, Ann Clin Exp Metabol 2017 [46]	Thin fibrous pseudo capsule, with areas of hemorrhage and necrosis areas	Interlacing fascicles of glycogen-rich (PAS-positive), spindle-shaped cells with moderate nuclear pleiomorphism.	Ki67 up to 30%; mitotic rate 7 to 8/10 HPFs	Positive for vimentin, actin, desmin, HHF35 and caldesmon; cytokeratin AE1 AE 3, HMB45, CD34, c-kit and S100 negative
MULANI SR, Case Rep Gastrointest Med 2018 [47]	-	Spindle cell neoplasm with extensive necrosis	High power view showing fascicular growth pattern and cytological pleomorphism.	Positive for desmin, cytokeratin Mak6, WT1, and S-100. Negative for CD34 and chromogranin
DOPPALAPUDI SK, BMJ Case Rep CP. 2019 [48]	Multilobular mass, without identified tissue plane	Hypercellular spindle cell neoplasm, cigar-shaped, running in parallel fascicles to one another, and demonstrated small, prominent nucleoli	-	Strong diffuse immunoreactivity to SMA and caldesmon
NERLI R, Int Cancer Confer J. 2020 [49]	Nodular, firm, well encapsulated tumor	Spindle-shaped tumor cells with prominent, eosinophilic cytoplasm resembling smooth muscle fibers	MIB 1/ki67 18–20% in the proliferative area of the tumor; nuclear mitosis increased	Positive for desmin and H caldesmon; ckit negative
SAKELLARIOU M, Mol Clin Oncol. 2020 [50]	Lobulated appearance, cystic degeneration, necrosis in cross section and residual adrenal tissue on outer tissue 594 g	Spindle cell tumor with intermediate nuclear atypia and small areas of ischemic necrosis	7 mitoses/10 HPFs; ki67 approximately 75%	Positive for desmin, SMA; negative for S100, synaptophysin, chromogranin, pan-cytokeratin, calretinin
LOKANATHA Indian J Med Paediatr Oncol. 2020 [51]	Encapsulated solid mass, roundish, with areas of hemorrhage	Spindle cell neoplasm	5–6 mitosis/10 HPFs; ki67 was 40%	Positive for SMA, desmin, H caldesmon and CD34, negative for CD117, S100, DOG1
Waack A, Radiol Case Rep. 2022 [1]	Well encapsulated adrenal mass with areas of necrosis	-	Increased of ki67	Positive for SMA, desmin, calponin, caldesmon and vimentin; negative for S100, CD34, ckit
Our Case Rep	Solid neoplastic proliferation	Spindle cells arranged in a fascicular pattern with areas of coagulative necrosis and areas of hyalinization of the stroma	Frequent mitotic figures	Positivity for SMA, desmin and calponin; negativity for cytokeratin, S100, EMA

α-SMA (alpha smooth muscle actin), HHF35 (muscle-actin-specific monoclonal antibody), HPFs (high power fields), HMWK (high molecular weight keratin), ALK (negative anaplastic lymphoma), (EMA) Epithelial Membrane Antigen.

## Data Availability

The datasets used and/or analyzed during the current study are available from the corresponding author on reasonable request.
